# Editorial Note: Can digital transformation promote green innovation in enterprises? The moderating effect of heterogeneous environmental regulations

**DOI:** 10.1371/journal.pone.0341490

**Published:** 2026-01-23

**Authors:** The PLOS One Editors

Following the publication of the Expression of Concern on this article [1,2], the corresponding author contacted PLOS to share the individual-level data underlying the results reported in the article. The *PLOS One* Editors issue this Editorial Note to provide an update to the Expression of Concern previously issued on this article and to provide the underlying data as S1 and S2 Files. The article’s [1] Data Availability statement is updated to: All relevant data are within the paper and its Supporting Information files. PLOS considers the data availability concerns resolved.

In light of the peer review concerns, the Expression of Concern stands.

## Supporting information

S1 File Regression data. The primary regression data used in the article to verify the main effect analysis and the moderated mediation effect analysis, specifically corresponding to Models 1, 2, 3, 4, and 5 in the paper. The specific results in this dataset correspond to Tables 3–7 in the article.
(XLSX)

S2 File Robustness test with reduced sample size. The specific validation results from this dataset are reported in Table 8.
(XLSX)
